# Aging Reshapes γ/δ T‐Cell Immunity Through a Type I Interferon–Foxo1 Axis

**DOI:** 10.1111/acel.70389

**Published:** 2026-01-20

**Authors:** Aurélie Durand, Sarah Porte, Eryang Xing, Christian Wu, Agnès Le Bon, Cédric Auffray, Bruno Lucas, Bruno Martin

**Affiliations:** ^1^ Université Paris‐Cité, Institut Cochin, Centre National de la Recherche Scientifique (CNRS) UMR8104, Institut National de la Santé et de la Recherche Médicale (INSERM) U1016 Paris France

**Keywords:** aging, Foxo1, type I IFNs, γ/δ T cells

## Abstract

Aging is associated with profound alterations in immune cell composition and function, yet the impact on peripheral γ/δ T‐cell subsets remains incompletely understood. Here, we show that the peripheral γ/δ T‐cell compartment is markedly remodeled with age in mice. Specifically, innate‐like Ly‐6C^−^ CD44^hi^ γ/δ T cells expand in secondary lymphoid organs (SLOs) of aged mice, while adaptive‐like subsets decline. This age‐related shift is accompanied by enhanced functionality, with Ly‐6C^−^ CD44^hi^ γ/δ T cells from aged SLOs displaying increased IL‐17 production both ex vivo and in vivo following LPS challenge. Mechanistically, this functional remodeling correlates with a significant decrease in the expression of the transcription factor Foxo1 in Ly‐6C^−^ CD44^hi^ γ/δ T cells. Type I interferon signaling contributes to the age‐dependent downregulation of Foxo1, as Ly‐6C^−^ CD44^hi^ γ/δ T cells from aged mice lacking the IFN‐α receptor maintain Foxo1 expression and exhibit reduced IL‐17 production. Collectively, our findings reveal that aging, through type I interferon–driven modulation of Foxo1, promotes the expansion and enhanced pro‐inflammatory activity of innate‐like γ/δ T cells. These changes may reinforce immune surveillance in secondary lymphoid organs but could also contribute to age‐associated immune dysregulation and inflammation.

## Graphical abstract

Aging reshapes the peripheral γ/δ T‐cell compartment by promoting the expansion and enhanced IL‐17–driven activity of innate‐like γ/δ T cells in secondary lymphoid organs. This remodeling is controlled by type I interferon–dependent downregulation of Foxo1, linking aging to heightened pro‐inflammatory γ/δ T‐cell responses.

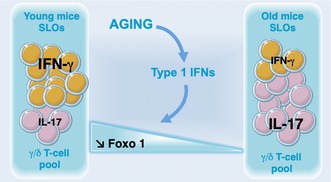

## 1 Introduction

Aging is a complex process that impacts multiple physiological functions, including the immune system. Indeed, aging significantly impairs immune surveillance, a crucial mechanism for preventing infectious diseases and tumor escape, leading to higher rates of illness and death (Kline and Bowdish 2016; Bottazzi et al. 2018; Drijvers et al. 2020). Additionally, aging diminishes the protective effectiveness of vaccination (Lefebvre et al. 2016; Allen et al. 2020). All these observations are the result of age‐related weakening of both the innate and adaptive immune systems (Mogilenko et al. 2022).

γ/δ T cells form a distinct subset of lymphocytes, differing from NK cells, B cells, and α/β T cells by combining adaptive properties with rapid, innate‐like responses (Dranoff 2004). This unique dual capacity allows them to play a crucial role throughout the immune response. The remarkable heterogeneity of the γ/δ T‐cell compartment is reflected in its diverse roles across immune responses. While their function in cancer immunity varies depending on tumor type and microenvironment (Lafont et al. 2014; Coffelt et al. 2015; Silva‐Santos et al. 2015), γ/δ T cells are known to detect cellular stress and contribute to tumor surveillance, as demonstrated in multiple murine models (Street et al. 2004; Girardi et al. 2001; Gao et al. 2003; Lança et al. 2013). Beyond their involvement in cancer, these cells are also strongly linked to autoimmune diseases such as multiple sclerosis (Sutton et al. 2009), rheumatoid arthritis (Roark et al. 2007; Keystone et al. 1991), and inflammatory bowel disease (Nanno et al. 2008; Mombaerts et al. 1993). Additionally, γ/δ T cells serve as key players in host defense against infections (Chien et al. 2014), highlighting their unique position at the intersection of innate and adaptive immunity.

The transcription factors of the forkhead box O (Foxo) family, particularly Foxo1, play a pivotal role in regulating α/β T‐cell key cellular processes, including quiescence, survival, trafficking, differentiation, and apoptosis (Gui and Burgering 2022; Kerdiles et al. 2009, 2010; Ouyang et al. 2010; Stone et al. 2015; Lainé et al. 2015; Malik and Awasthi 2018). Interestingly, we recently found that the age‐related decline in Foxo1 expression in mouse α/β T cells may drive the disruption of their peripheral homeostasis and contribute to the aging of this T‐cell compartment (Durand et al. 2024).

Taken together, all these findings prompted us to investigate how the functional capacities of γ/δ T cells are affected by aging, as well as whether Foxo1 expression could be modulated in this T‐cell compartment with age. Therefore, in this study, we demonstrate that, as observed for α/β T cells, the homeostasis of the peripheral γ/δ T‐cell compartment is markedly altered with age. Indeed, a comparison of the γ/δ T‐cell compartment within the SLOs of old (18‐month‐old) and young (3‐month‐old) adult mice reveals that aging promotes the expansion of innate‐like γ/δ T cells and enhances their capacity to produce IL‐17. Notably, we found that these age‐related changes were associated with the loss of Foxo1 expression within this T‐cell compartment. Finally, as observed in α/β T cells, our results indicate that the age‐related decline in Foxo1 expression in γ/δ T cells is likely driven by a similar T cell‐extrinsic factor. In this context, we identify type I IFNs as a key regulator that down‐regulates Foxo1 in IL‐17‐producing γ/δ T cells with age and enhances the capacity of Ly‐6C^−^ CD44^hi^ γ/δ T lymphocytes to mount a rapid in vivo response to LPS challenge during aging.

## 2 Materials and Methods

### 2.1 Mice

3‐month‐old and 18‐month‐old mice were used for experiments. C57BL/6 WT and C57BL/6 Ifnar^KO^ mice (Müller et al. 1994; Taleb et al. 2017) were maintained in our own animal facilities, under specific pathogen–free (SPF) conditions. The old mice and their young adult controls were all female mice. Mice were housed in separate individually ventilated cages in the same controlled environment (12 h‐light–dark cycle, 22°C ± 2°C, 30%–70% humidity), with standard rodent chow and water ad libitum. All tests for rodent pathogens carried out during the 5 years we have been working on our aging colony have been negative. All procedures were approved by the ethics committee for animal experimentation n°34 and validated by the “Ministère de l'Enseignement Supérieur, de la Recherche et de l'Innovation” with the number APAFIS ≠ 20,630‐2018033016303981v5. Sample sizes were chosen to ensure reproducibility of the experiments and in accordance with the 3R of animal ethics regulation.

### 2.2 Cell Suspensions

All mice were sacrificed by vertebral dislocation and peripheral lymph‐nodes (pooled cervical, axillary, brachial, and inguinal lymph‐nodes; pLNs), mesenteric LNs (mLNs), spleen, and thymus were harvested and homogenized, and passed through a nylon cell strainer (BD Falcon) in RPMI 1640 GlutaMAX (Life Technologies) supplemented with 10% FCS (Biochrom) for determination of intracellular cytokine production or in 5% FCS, 0.1% NaN3 (Interchim) in phosphate buffered saline (PBS) for all the other flow cytometry experiments.

### 2.3 Cell Cultures

γ/δ T cells were purified from LNs (pooled superficial cervical, axillary, brachial, inguinal, and mLNs) of C57BL/6 mice by incubating cell suspensions on ice for 20 min with a mixture of anti‐CD11b (Mac‐1, Biolegend), anti‐CD19 (1D3, BioXcell), anti‐Ter‐119 (TER‐119, Biolegend), anti‐CD4 (GK1.5) and anti‐CD8α (53–6.7) (anti‐CD4 and anti‐CD8α Abs were obtained from hybridoma supernatants) and then with magnetic beads coupled to anti‐rat Igs (Dynal Biotech). Then, γ/δ T cells were sorted by flow cytometry as TCRγ/δ^+^ Lineage^−^ (NK1.1^−^ CD19^−^ CD11b^−^ CD11c^−^) cells using a FACSAria III flow cytometer (BD Biosciences). Finally, 10^5^ cells were cultured in the presence of IL‐7 (10 ng/mL; R&D Systems) alone or together with IFNα4 (10^4^ U/mL (Taleb et al. 2017)), Alpelisib (PI3Kinhibitor; 5 μM; MedChemExpress) and AKT inhibitor VIII (AKTi; 0.75 μM; Calbiochem).

### 2.4 Fluorescence Staining and Flow Cytometry

Cell suspensions were collected and dispensed into 96‐well round‐bottom microtiter plates (Greiner Bioscience; 6 × 10^6^ cells/well). Surface staining was performed as previously described. Briefly, cells were incubated on ice for 15 min per step with antibodies (Abs) in 5% FCS (Biochrom), 0.1% NaN_3_ (Sigma‐Aldrich) PBS. Each cell‐staining reaction was preceded by a 15‐min incubation with a purified anti‐mouse CD16/32 Ab (FcγRII/III block; 2.4G2, BioXcell). The Foxp3 Staining Buffer Set (eBioscience) was used for Foxo1 intracellular staining. Briefly, cells were barcoded using anti‐CD45 antibodies and stained for cell surface antigens. After an additional wash, intracellular Foxo1 was stained overnight at 4°C. For determination of intracellular cytokine production, cells were stimulated with 0.5 μg/mL PMA (Sigma‐Aldrich), 0.5 μg/mL ionomycin (Sigma‐Aldrich), and 10 μg/mL BrefeldinA (Sigma‐Aldrich) for 2 h at 37°C. Cells were then stained for surface markers, fixed in 2% paraformaldehyde in PBS, and permeabilized with 0.5% saponin, followed by labeling with cytokine‐specific Abs. Multi‐color immunofluorescence was analyzed using a BD‐Fortessa cytometer (BD Biosciences). Data acquisition was performed at the Cochin CYBIO facility. List of used antibodies is shown in Table S1.

### 2.5 In Vivo LPS Challenge

Young (3‐month‐old) and old (18‐month‐old) C57BL/6 WT mice or old (18‐month‐old) C57BL/6 Ifnar^KO^ mice were injected intravenously with 100 μg of LPS (from *Escherichia coli* 0111:B4; Sigma). 3 h after LPS challenge, cells recovered from pLNs, mLNs, and the spleen were incubated with 10 μg/mL BrefeldinA (Sigma‐Aldrich) for 2 h at 37°C for determination of intracellular cytokine production.

### 2.6 Single‐Cell RNA‐Seq Data Processing and Analysis of γ/δ T Cells

Publicly available scRNA‐sequencing datasets from the recent study of Zhang et al. (2025) were reanalyzed. γ/δ T cells from lung, ileum, and colon were extracted and analyzed independently for each tissue. All analyses were performed in R (version 4.5.1) using Seurat (version 5.3.1), along with additional packages including dplyr, ggplot2, clustree, UCell, SmoothKNN, patchwork, and ggridges.

#### 2.6.1 Quality Control and Filtering

For each tissue, cells were filtered based on standard quality control criteria. Cells with fewer than 200 detected genes, an excessive number of detected genes (indicative of potential doublets; tissue‐specific upper thresholds were applied), or a mitochondrial gene content greater than 5% were excluded. Mitochondrial gene percentages were calculated using genes matching the pattern “^mt‐”.

#### 2.6.2 Normalization and Dimensionality Reduction

Filtered datasets were normalized using the SCTransform method implemented in Seurat, regressing out the percentage of mitochondrial gene expression. Highly variable genes (up to 5000 per dataset) were selected automatically by SCTransform. Principal component analysis (PCA) was performed on the SCT‐normalized data. The number of principal components retained for downstream analyses was determined based on the cumulative variance explained (> 90%) and the inflection point in the variance distribution across components.

#### 2.6.3 Clustering and Visualization

Graph‐based clustering was performed using the shared nearest neighbor (SNN) approach implemented in Seurat, across a range of clustering resolutions. Final clustering resolutions were selected based on cluster stability assessed using clustree. Uniform Manifold Approximation and Projection (UMAP) was used for two‐dimensional visualization of the data. Clusters were visualized across age groups and sex to assess age‐ and sex‐associated changes in γ/δ T‐cell composition within each tissue.

#### 2.6.4 Gene Expression and Signature Analyses

Cluster identities were characterized using the expression of known γ/δ T‐cell–associated markers, visualized using dot plots. To reduce local noise and improve the robustness of signature scores, UCell scores were smoothed using SmoothKNN based on the SCT PCA space. Transformed signature scores (arcsinh) were used for visualization.

#### 2.6.5 Statistical Representation of Age‐Associated Changes

Age‐ and sex‐associated changes in γ/δ T‐cell cluster frequencies were quantified as percentages relative to the total number of cells profiled per age group in each tissue. Temporal dynamics were visualized using line plots.

### 2.7 Statistical Analysis

Data are expressed as mean ± SEM, and the significance of differences between two series of results was assessed using the Student's unpaired *t* test (*p < 0.05; **p < 0.01; ***p < 0.001; ****p < 0.0001, ns, not significant).

### 2.8 Software for Data Collection and Analysis

Flow Cytometry data were analyzed using DIVA 8.0.1 (BD Biosciences), Prism 8.2.1 (GraphPad), and Excel 15.29.1 (Microsoft).

## 3 Results

### 3.1 The Peripheral γ/δ T‐Cell Compartment Is Markedly Altered With Age

**FIGURE 1:**
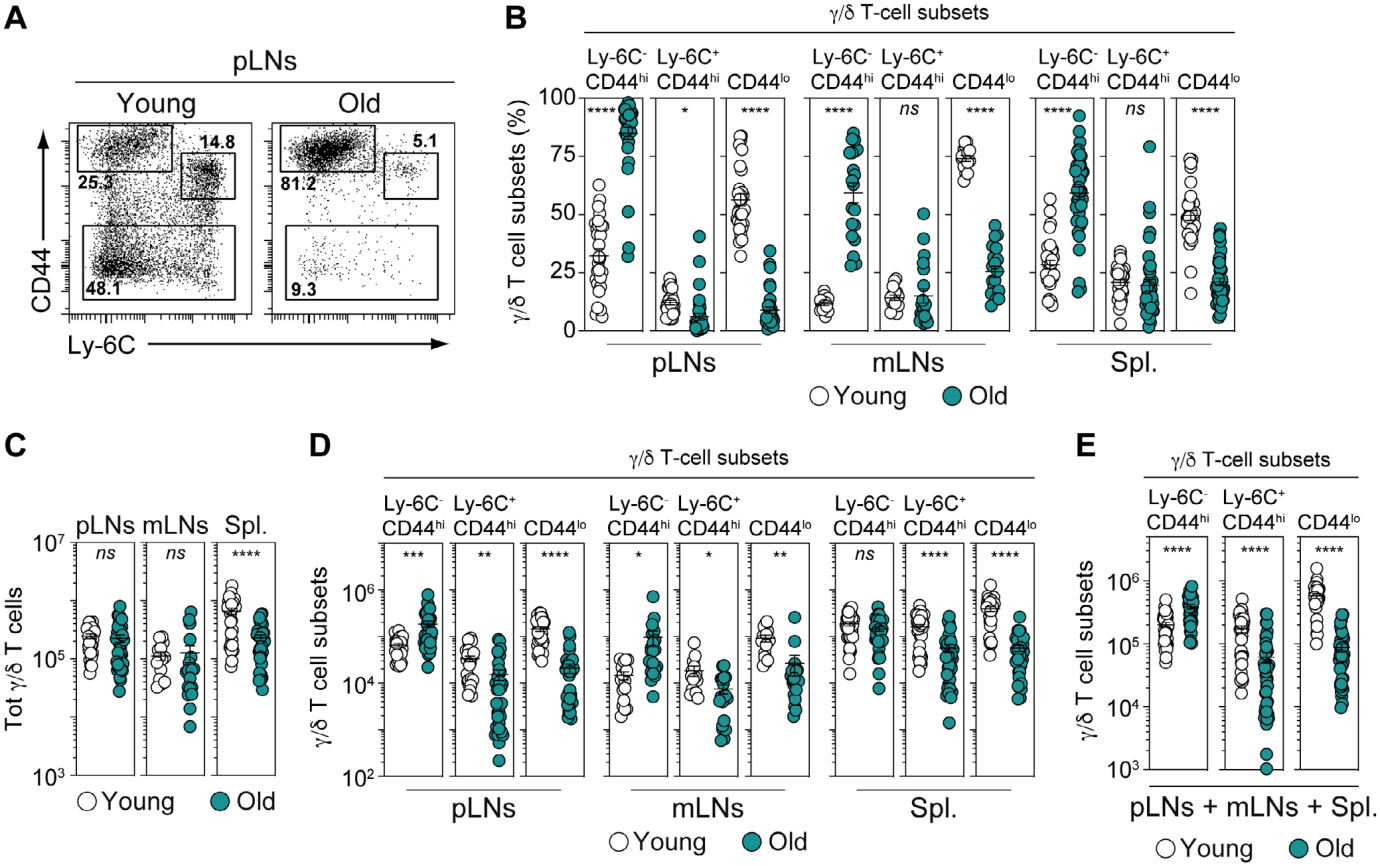
The peripheral γ/δ T‐cell compartment is markedly altered with age. (A) CD44/Ly‐6C dot‐plots for gated TCRγ/δ^+^ cells recovered from pLNs of representative young (3‐month‐old) and old (18‐month‐old) adult C57BL/6 mice. Numbers on FACS dot‐plots indicate the percentage of each γ/δ T‐cell subset among the γ/δ T lymphocyte compartment. (B) Percentage of γ/δ T‐cell subsets, classified by Ly‐6C and CD44 expression, recovered from pLNs, mLNs and spleen of young (3‐month‐old) and old (18‐month‐old) adult C57BL/6 mice. (C) Absolute numbers of total γ/δ T cells recovered from peripheral LNs (pLNs), mesenteric LNs (mLNs) and spleen (Spl.) of young (3‐month‐old) and old (18‐month‐old) adult C57BL/6 mice. (D) Absolute numbers of γ/δ T‐cell subsets, categorized by their Ly‐6C and CD44 expression, recovered from pLNs, mLNs and the spleen of young (3‐month‐old) and old (18‐month‐old) adult C57BL/6 mice. (E) Total (pLNs + mLNs + Spl.) absolute numbers of the different γ/δ T‐cell subsets, categorized by their Ly‐6C and CD44 expression, recovered from young (3‐month‐old) and old (18‐month‐old) adult C57BL/6 mice. Data are expressed as mean ± SEM. Results, from at least three independent experiments, were assessed using a two‐tailed unpaired Student's *t*‐test (*p < 0.05; **p < 0.01; ***p < 0.001; ****p < 0,0001; ns, not significant). Each dot on figure panels represents individual mice.

According to Ly‐6C and CD44 expression, we have documented the heterogeneity of the peripheral γ/δ T‐cell compartment by showing that it comprises Ly‐6C^−^ CD44^hi^ γ/δ T cells exhibiting innate‐like features and naive and memory adaptive‐like γ/δ T cells Ly6C^+/−^ CD44^lo^ γ/δ T cells and Ly‐6C^+^ CD44^hi^ γ/δ T cells respectively (Lombes et al. 2015; Audemard‐Verger et al. 2017). To go further, we have now decided to study the impact of aging in the homeostasis of these peripheral T‐cell subsets. For this purpose, we performed a qualitative and quantitative comparison of the peripheral γ/δ T‐cell compartment recovered from young (3‐month‐old) and old (18‐month‐old) adult mice. We initially observed that the proportion of Ly‐6C^−^ CD44^hi^ γ/δ T cells was greatly increased in all studied secondary lymphoid organs (SLOs) of aged mice (Figure 1A,B). In contrast, the percentages of adaptive‐like γ/δ T‐cell subsets, particularly CD44ˡ° γ/δ T cells, were markedly reduced. Quantitatively, we observed that the total number of γ/δ T cells was reduced in the spleen of aged mice, while it remained unchanged in peripheral or mesenteric lymph nodes (Figure 1C). However, this apparent stability, particularly in lymph nodes, hides significant qualitative differences. Indeed, in both peripheral and mesenteric lymph nodes, we noticed a marked increase in the absolute number of innate‐like Ly‐6C^−^ CD44^hi^ γ/δ T cells, along with a significant decrease in adaptive‐like γ/δ T‐cell subsets (Figure 1D). Consistent with these results, by summing the absolute numbers of the different γ/δ T‐cell subsets recovered from peripheral and mesenteric lymph nodes and the spleen, we observed an overall increase in innate‐like Ly‐6C^−^ CD44^hi^ γ/δ T cells and a decrease in adaptive‐like γ/δ T‐cell populations (Figure 1E). Given the tissue‐resident nature of γ/δ T cells, we next examined whether the age‐associated changes observed in SLOs were accompanied by similar alterations in γ/δ T‐cell populations in non‐lymphoid tissues, or whether they could result from a redistribution of tissue‐resident γ/δ T cells. To do so, we reanalyzed publicly available single‐cell RNA‐sequencing datasets from a recently published study (Zhang et al. 2025), which provides a comprehensive, multi‐organ characterization of immune cell populations across the mouse lifespan (Figure S1). This analysis enabled us to define γ/δ T‐cell clusters in the lung, colon, and ileum of C57BL/6 mice (Figure S1A). Examination of cluster‐specific gene expression signatures, including transcription factors such as Rora and Rorc, and cytokine receptors for IL‐1, IL‐7, and IL‐23, allowed the identification of clusters corresponding to innate‐like γ/δ T cells in the lung (clusters 1 and 3) as well as in the colon and ileum (clusters 1 and 2) (Figure S1B). We then analyzed age‐associated changes in the relative proportions of the defined γ/δ T‐cell clusters within these tissues. While the overall proportions of γ/δ T‐cell clusters appeared largely stable with age in the lung, we observed an age‐related increase in innate‐like γ/δ T cells in the colon and ileum that was restricted to cluster 1 (Figure S1C). Notably, this cluster is composed of cells expressing high levels of the IL‐7 receptor (Figure S1B). As reported by previous studies (Wencker et al. 2014; Chen et al. 2019), this elevated IL‐7R expression makes these cells phenotypically resemble the innate‐like γ/δ17 T‐cell lineage present in SLOs. Altogether, these findings indicate that aging profoundly reshapes the homeostasis of the γ/δ T‐cell compartment in SLOs, driving the expansion of Ly‐6C^−^ CD44^hi^ innate‐like γ/δ T cells along with a marked loss in adaptive‐like γ/δ T‐cell subsets in SLOs, and suggest that this process does not result from the redistribution of non‐lymphoid tissue‐resident γ/δ T‐cell subsets.

### 3.2 Aging Enhances the Capacity of Ly‐6C ^−^ CD44^hi^ γ/δ T Cells to Produce IL‐17 in SLOs

**FIGURE 2:**
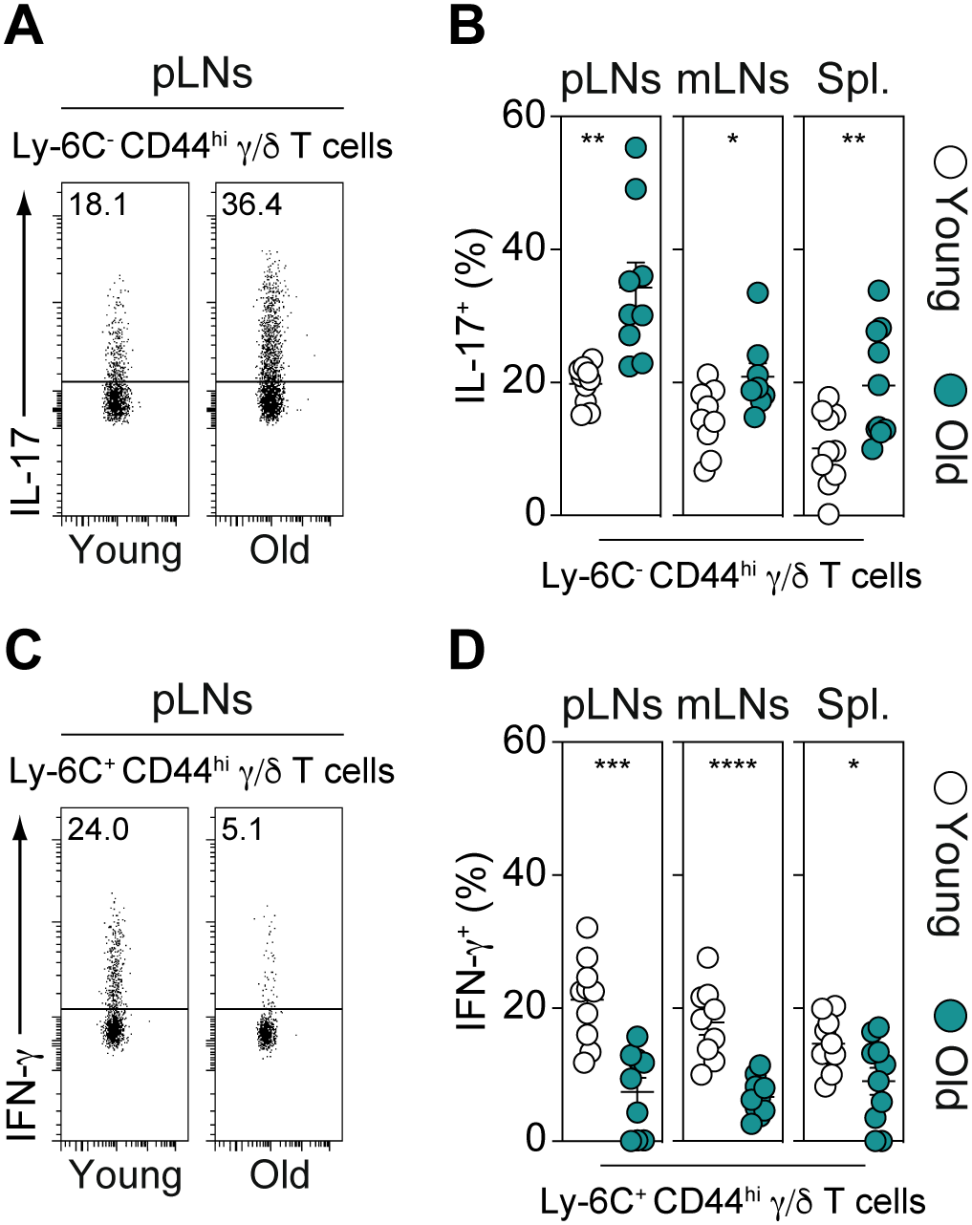
Aging enhances the capacity of Ly‐6C^−^ CD44^hi^ γ/δ T cells to produce IL‐17 in SLOs. To assess their cytokine production capacity, γ/δ T cells recovered from peripheral LNs (pLNs), mesenteric LNs (mLNs), and the spleen (Spl.) of young (3‐month‐old) and old (18‐month‐old) adult C57BL/6 mice were ex vivo stimulated with PMA, ionomycin, and BrefeldinA for 2 h. (A) Representative IL‐17 dot‐plots for gated Ly‐6C^−^ CD44^hi^ γ/δ T cells recovered from pLNs of young (3‐month‐old) and old (18‐month‐old) adult C57BL/6 mice. Numbers on FACS dot‐plots indicate the percentage of IL‐17^+^ cells among Ly‐6C^−^ CD44^hi^ γ/δ T cells. (B) Proportions of IL‐17‐producing cells among Ly‐6C^−^ CD44^hi^ γ/δ T cells recovered from pLNs, mLNs, and spleen of young (3‐month‐old) and old (18‐month‐old) adult C57BL/6 mice. (C) Representative IFN‐γ dot‐plots for gated Ly‐6C^+^ CD44^hi^ γ/δ T cells recovered from pLNs of young (3‐month‐old) and old (18‐month‐old) adult C57BL/6 mice. Numbers on FACS dot‐plots indicate the percentage of IFN‐γ^+^ cells among Ly‐6C^+^ CD44^hi^ γ/δ T cells. (D) Proportions of IFN‐γ‐producing cells among Ly‐6C^+^ CD44^hi^ γ/δ T cells recovered from pLNs, mLNs, and spleen of young (3‐month‐old) and old (18‐month‐old) adult C57BL/6 mice. Data are expressed as mean ± SEM. Results, from at least three independent experiments, were assessed using a two‐tailed unpaired Student's *t*‐test (*p < 0.05; **p < 0.01; ***p < 0.001; ****p < 0,0001, ns, not significant). Each dot on figure panels represents individual mice.

In addition to their phenotypic heterogeneity, we previously reported that innate‐like Ly‐6C^−^ CD44^hi^ γ/δ T cells and adaptive‐like γ/δ T‐cell subsets from SLOs exhibit distinct functional capacities. Indeed, while Ly‐6C^−^ CD44^hi^ γ/δ T cells specifically produce IL‐17, the other γ/δ T‐cell subsets are predominantly oriented toward IFN‐γ production (Lombes et al. 2015). Consequently, we then examined whether aging could affect the functional capacities of γ/δ T cells, particularly their cytokine production ability. To do so, we compared the capacity of these T‐cell subsets, recovered from SLOs of young or old mice, to produce IL‐17 or IFN‐γ following ex vivo re‐stimulation (Figure 2). As shown in Figure 2A,B, we found that the proportion of Ly‐6C^−^ CD44^hi^ γ/δ T cells capable of producing IL‐17 was significantly increased with age in all studied SLOs. By contrast, in this setting, the ability of Ly‐6C^+^ CD44^hi^ γ/δ T cells to produce IFN‐γ markedly diminished with aging (Figure 2C,D). These findings indicate that aging not only promotes the expansion of Ly‐6C^−^ CD44^hi^ innate‐like γ/δ T cells in SLOs but also enhances their capacity to produce IL‐17.

### 3.3 The Capacity of Ly‐6C ^−^ CD44^hi^ γ/δ T Lymphocytes From SLOs to Mount a Rapid In Vivo Response Upon LPS Challenge Increases With Age

**FIGURE 3:**
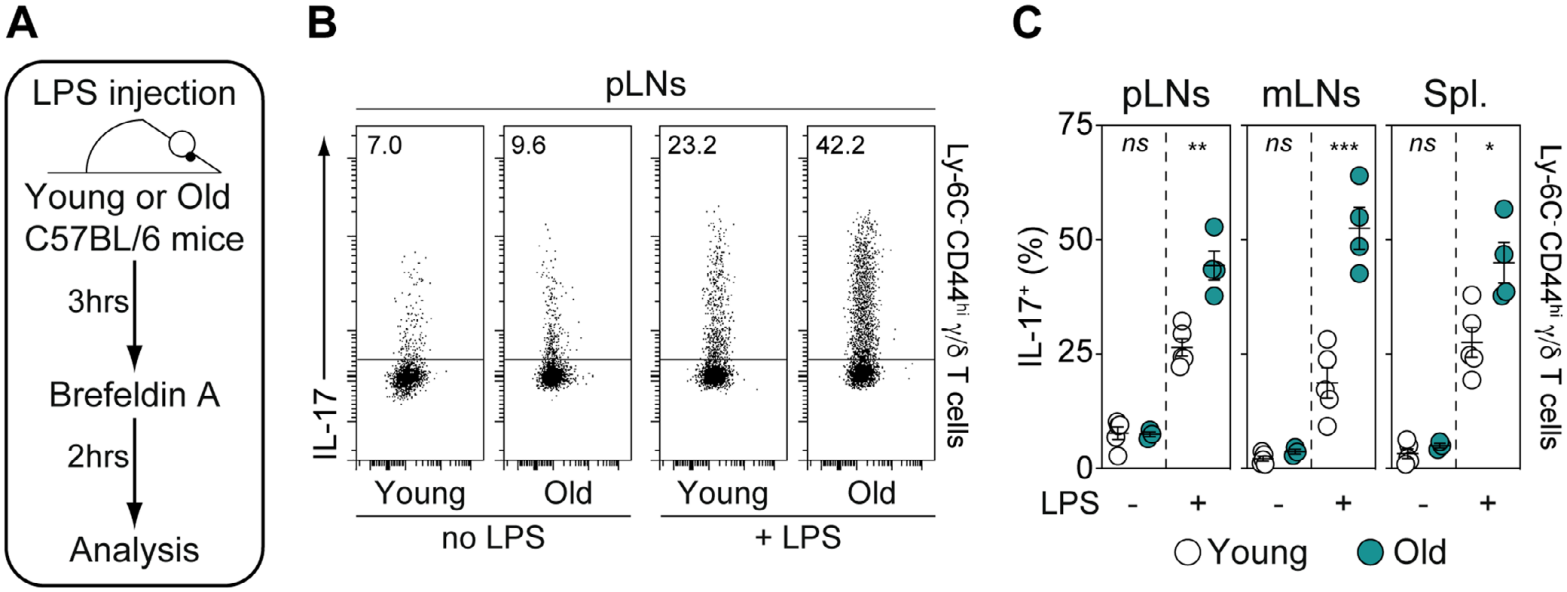
The capacity of Ly‐6C^−^ CD44^hi^ γ/δ T lymphocytes from SLOs to mount a rapid in vivo response upon LPS challenge increases with age. Young (3‐month‐old) and old (18‐month‐old) adult C57BL/6 mice were injected i.v. with LPS. 3 h later, cells recovered from peripheral LNs (pLNs), mesenteric LNs (mLNs) and the spleen (Spl.) were incubated during 2 h with BrefeldinA for determination of intracellular cytokine production. (A) Diagram illustrating the experimental model. (B) Representative IL‐17 dot‐plots for gated Ly‐6C^−^ CD44^hi^ γ/δ T cells recovered from pLNs of LPS‐treated or ‐untreated young (3‐month‐old) and old (18‐month‐old) adult C57BL/6 mice. (C) Proportions of IL‐17‐producing cells among Ly‐6C^−^ CD44^hi^ γ/δ T cells recovered from pLNs, mLNs, and spleen of LPS‐treated or ‐untreated young (3‐month‐old) and old (18‐month‐old) adult C57BL/6 mice. Data are expressed as mean ± SEM. Results, from at least three independent experiments, were assessed using a two‐tailed unpaired Student's *t*‐test (*p < 0.05; **p < 0.01; ***p < 0.001; ****p < 0,0001, ns, not significant). Each dot on figure panels represents individual mice.

We and others have previously described that Ly‐6C^−^ CD44^hi^ γ/δ T lymphocytes establish strong adhesive interactions with macrophages, which may contribute to their long‐term retention within SLOs (Audemard‐Verger et al. 2017; Zhang et al. 2016). Additionally, we found that Ly‐6C^−^ CD44^hi^ γ/δ T cells rapidly produce IL‐17 upon in vivo LPS challenge, suggesting that these SLO‐resident γ/δ T cells play a crucial role in immune‐surveillance, protecting these organs from infections (Audemard‐Verger et al. 2017). To further investigate, we analyzed whether aging modulates the capacity of this innate‐like γ/δ T‐cell subset to respond to LPS challenge. To address this issue, young (3‐month‐old) and old (18‐month‐old) adult mice were intravenously injected with LPS. 3 h later, IL‐17 production by Ly‐6C^−^ CD44^hi^ γ/δ T cells recovered from pLNs, mLNs and the spleen was analyzed (Figure 3A). We observed that in all SLOs, the proportion of these cells producing IL‐17 in response to LPS was strongly increased with age (Figure 3B,C). These results suggest that aging may enhance the immune‐surveillance functions of SLO‐resident Ly‐6C^−^ CD44^hi^ γ/δ T cells.

### 3.4 Foxo1 Expression Is Strongly Down‐Regulated in Ly‐6C ^−^ CD44^hi^ γ/δ T Lymphocytes With Age

**FIGURE 4:**
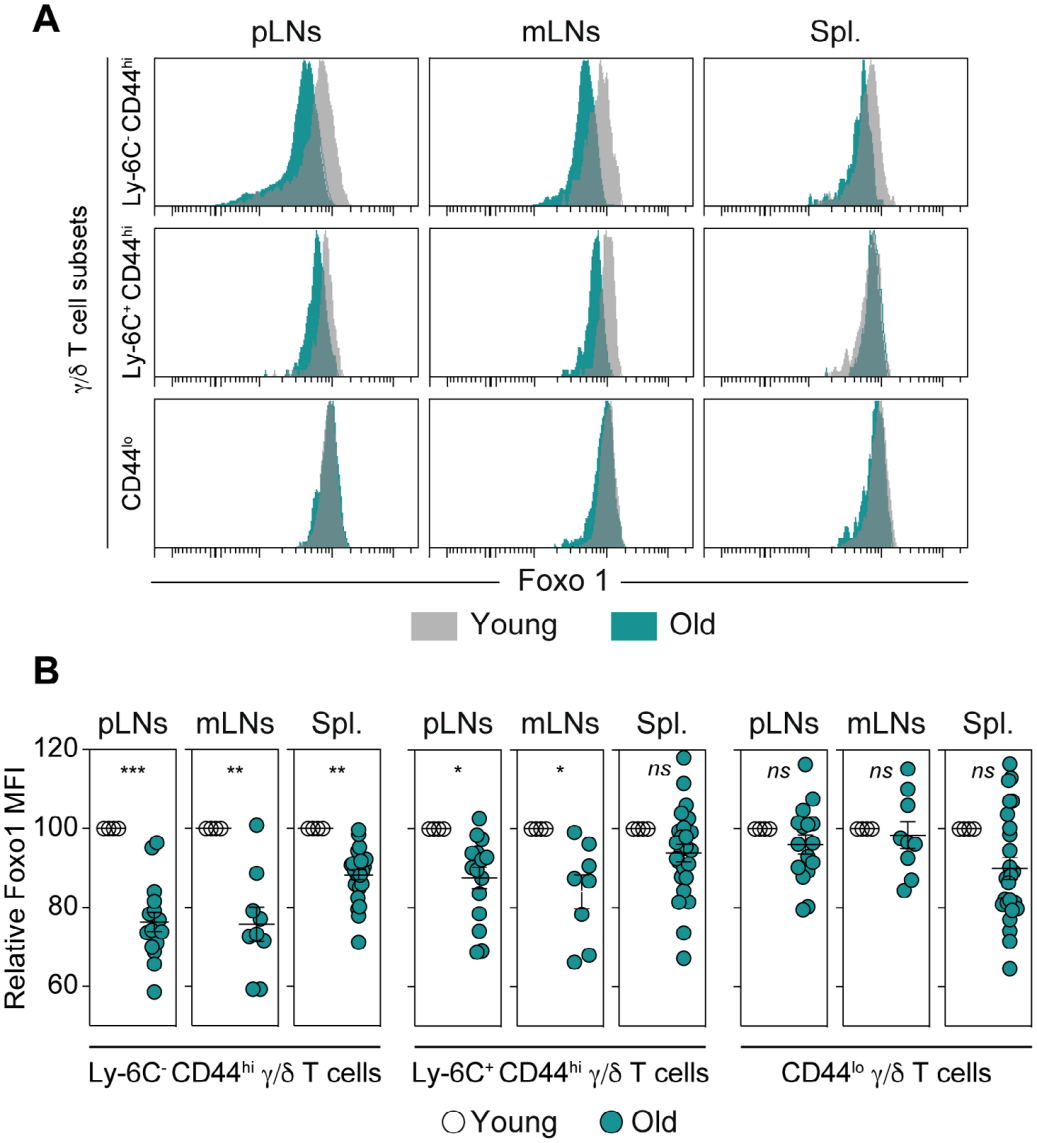
Foxo1 expression is highly down‐regulated in Ly‐6C^−^ CD44^hi^ γ/δ T lymphocytes with age. Cell suspensions from peripheral LNs (pLNs), mesenteric LNs (mLNs), and the spleen (Spl.) of young (3‐month‐old) and old (18‐month‐old) adult C57BL/6 mice were first stained separately with anti‐CD45 antibodies conjugated to different fluorochromes and then mixed before further staining. (A) Foxo1 fluorescence histograms of the indicated γ/δ T‐cell subsets are shown for representative mice. (B) Relative Mean Fluorescence Intensities (MFIs) were calculated by dividing the MFI of a given γ/δ T‐cell subset of the old mouse by the MFI of the same γ/δ T‐cell subset of the barcoded young adult mouse. Results from at least three independent experiments were assessed using a two‐tailed unpaired Student's *t*‐test (*p < 0.05; **p < 0.01; ***p < 0.001; ****p < 0,0001; ns, not significant). Each dot on figure panels represents individual mice.

We previously described that the transcription factor Foxo1 acts as a direct negative regulator of the RORγt‐Th17 differentiation program in CD4 α/β T cells (Lainé et al. 2015). Additionally, we recently observed that Foxo1 expression is down‐regulated with age in mouse α/β T cells (Durand et al. 2024). Thus, we investigated whether the age‐related increase in IL‐17 production by γ/δ T cells correlates with the down‐regulation of Foxo1 expression in these cells. To this end, we next compared Foxo1 expression in peripheral γ/δ T‐cell subsets from young and old adult mice. For this purpose, cell suspensions from SLOs of young and old adult mice were first labeled separately with anti‐CD45 antibodies conjugated to different fluorochromes and then mixed together so that surface and intracellular staining were performed in a single well (Durand et al. 2024). Interestingly, Foxo1 expression decreased significantly in Ly‐6C^−^ CD44^hi^ γ/δ T cells recovered from all studied SLOs with age (Figure 4A,B). By contrast, Foxo1 expression was only slightly decreased in Ly‐6C^+^ CD44^hi^ γ/δ T cells from peripheral and mesenteric LNs, while it remained unaffected in CD44^lo^ γ/δ T cells (Figure 4A,B). Altogether, these results indicate that Foxo1 expression is strongly down‐regulated in peripheral Ly‐6C^−^ CD44^hi^ γ/δ T lymphocytes with age, correlating with their increased capacity to produce IL‐17.

### 3.5 Type I Interferons Drive Foxo1 Down‐Regulation in IL‐17‐Producing γ/δ T Cells With Age

**FIGURE 5:**
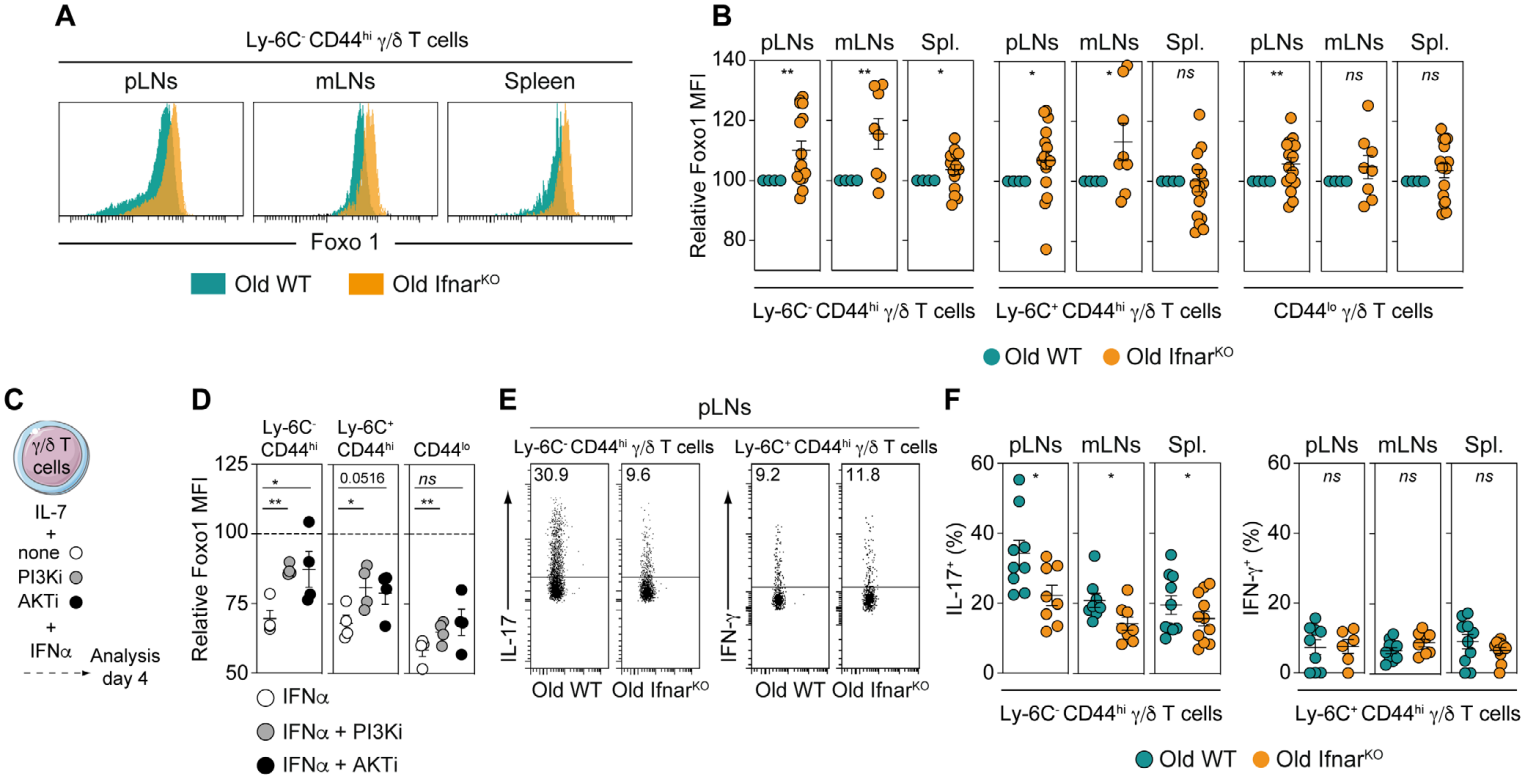
Type I interferons drive Foxo1 down‐regulation in IL‐17‐producing γ/δ T cells with age. (A, B) Cell suspensions from peripheral LNs (pLNs), mesenteric LNs (mLNs) and the spleen (Spl.) of old WT (18‐month‐old) and old Ifnar^KO^ (18‐month‐old) C57BL/6 mice were first stained separately with anti‐CD45 antibodies conjugated to different fluorochromes and then mixed before further staining. (A) Foxo1 fluorescence histograms of Ly‐6C^−^ CD44^hi^ γ/δ T cells are shown for representative mice. (B) Relative Mean Fluorescence Intensities (MFIs) were calculated by dividing the MFI of a given γ/δ T‐cell subset of the old Ifnar^KO^ mouse by MFI of the same γ/δ T‐cell subset of the barcoded old WT mouse. (C‐D) FACS‐sorted γ/δ T cells were cultured for 4 days in the presence of IL‐7 alone or with IFNα4 alone or in combination with PI3K or AKT inhibitors. (C) Diagram illustrating the experimental model. (D) Relative Foxo1 MFIs for the different γ/δ T‐cell subsets recovered after 4 days of culture with IL‐7 in the presence of IFNα4 and the indicated inhibitors. Relative MFIs were calculated after barcoding by dividing the MFI of a given γ/δ T‐cell subset in the presence of one given inhibitor and IFNα4 by the MFI of the same γ/δ T‐cell subset cultured with the same inhibitor alone. Statistics were calculated to compare Foxo1 down‐regulation induced by IFNα4 alone with Foxo1 down‐regulation induced by IFNα4 in the presence of the indicated inhibitors. Data are expressed as mean ± SEM. Each dot represents an individual experiment. Results were assessed using an unpaired Student's *t*‐test (*p < 0.05; **p < 0.01; ***p < 0.001; ****p < 0,0001, ns, not significant). (E, F) To assess their cytokine production capacity, γ/δ T cells recovered from peripheral LNs (pLNs), mesenteric LNs (mLNs) and the spleen (Spl.) of old (18‐month‐old) WT and old Ifnar^KO^ (18‐month‐old) C57BL/6 mice were ex vivo stimulated with PMA, ionomycin and BrefeldinA for 2 h. (E) Representative IL‐17 and IFN‐γ dot‐plots for gated Ly‐6C^−^ CD44^hi^ or Ly‐6C^+^ CD44^hi^γ/δ T cells recovered from pLNs of old WT and old Ifnar^KO^ C57BL/6 mice. (F) Proportions of IL‐17‐ or IFN‐γ‐producing cells among Ly‐6C^−^ CD44^hi^ or Ly‐6C^+^ CD44^hi^ γ/δ T cells recovered from pLNs, mLNs and spleen of old WT and old Ifnar^KO^ C57BL/6 mice. Data are expressed as mean ± SEM. Results, from at least three independent experiments, were assessed using a two‐tailed unpaired Student's *t*‐test (*p < 0.05; **p < 0.01; ***p < 0.001; ****p < 0,0001, ns, not significant). Each dot on figure panels represents individual mice.

In a recent study, we found that the age‐driven Foxo1 down‐regulation in α/β T cells is mediated by T‐cell‐extrinsic cues, notably type I interferons (Durand et al. 2024). We therefore examined whether type I IFNs could also be responsible for the age‐related down‐regulation of Foxo1 expression in peripheral Ly‐6C^−^ CD44^hi^ γ/δ T lymphocytes. To do so, we next compared Foxo1 expression in peripheral γ/δ T‐cell subsets from 18‐month‐old WT mice and 18‐month‐old mice deficient for the expression of the type I interferon receptor (mice knock‐out for the subunit 1 of the type I IFN receptor, Ifnar^KO^ mice (Müller et al. 1994)). Interestingly, Foxo1 expression was significantly higher in Ly‐6C^−^ CD44^hi^ γ/δ T cells recovered from all studied SLOs of aged Ifnar^KO^ mice compared to old WT mice (Figure 5A,B). Of note, Foxo1 expression was also significantly increased in adaptive‐like naïve CD44^lo^ γ/δ T cells from peripheral LNs and in memory Ly‐6C^+^ CD44^hi^ γ/δ T cells from both peripheral and mesenteric LNs of aged Ifnar^KO^ mice compared to old WT mice (Figure 5B). By contrast, no differences were observed between old WT and Ifnar^KO^ mice in the absolute numbers of the various γ/δ T‐cell subsets recovered from all studied SLOs (Figure S2), suggesting that the age‐associated expansion of innate‐like γ/δ T cells does not result from the decreased Foxo1 expression observed in these cells.

We recently found in vitro that type I IFNs induce Foxo1 downregulation in α/β T cells through a mechanism involving the PI3K‐AKT pathway (Durand et al. 2024). We then sought to determine whether this pathway could also contribute to the age‐related modulation of Foxo1 expression in peripheral γ/δ T cells. To do so, we cultured FACS‐sorted peripheral γ/δ T cells in the presence of IFN‐α alone or in combination with PI3K or AKT inhibitors for 4 days (Figure 5C). In this setting, we observed that IFN‐α induced a significant decrease in Foxo1 expression across all γ/δ T‐cell subsets (Figure 5D). Notably, at least for Ly‐6C^−^ CD44^hi^ and Ly‐6C^+^ CD44^hi^ γ/δ T cells, both PI3K and AKT inhibitors clearly attenuated the type I IFN‐induced downregulation of Foxo1 (Figure 5D). These findings suggest that, as reported for α/β T cells (Durand et al. 2024), type I IFN signaling may contribute to the age‐related modulation of Foxo1 expression in peripheral γ/δ T cells, potentially through the involvement of the PI3K‐AKT pathway.

Because type I IFNs induce significant down‐regulation of Foxo1 in γ/δ T‐cell subsets with age, we then examined whether type I IFNs could consequently affect the functional capacities of these cells, particularly their cytokine production ability. To do so, we compared the ability of both Ly‐6C^−^ CD44^hi^ and Ly‐6C^+^ CD44^hi^ γ/δ T‐cell subsets, recovered from the SLOs of aged Ifnar^KO^ and old WT mice, to produce IL‐17 or IFN‐γ respectively, following ex vivo re‐stimulation. As shown in Figure 5D,E, Ly‐6C^−^ CD44^hi^ γ/δ T cells exhibited a greater capacity to produce IL‐17 in all studied SLOs of old WT mice than in the SLOs of aged Ifnar^KO^ mice. By contrast, the ability of peripheral Ly‐6C^+^ CD44^hi^ γ/δ T cells to produce IFN‐γ was unaffected in Ifnar^KO^ mice with age (Figure 5D,E).

Taken together, these results suggest that type I IFNs may drive the age‐dependent down‐regulation of Foxo1 expression in peripheral γ/δ T cells, which in turn may enhance the capacity of the innate‐like Ly‐6C^−^ CD44^hi^ γ/δ T‐cell subset to produce IL‐17.

### 3.6 Type I Interferons Enhance the Ability of Ly‐6C ^−^ CD44^hi^ γ/δ T Lymphocytes to Mount a Rapid In Vivo Response to LPS Challenge With Age

**FIGURE 6:**
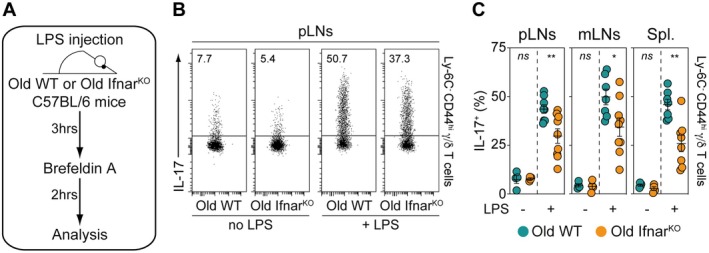
Type I interferons enhance the ability of Ly‐6C^−^ CD44^hi^ γ/δ T lymphocytes to mount a rapid in vivo response to LPS challenge with age. Old WT (18‐month‐old) and old Ifnar^KO^ (18‐month‐old) C57BL/6 mice were injected i.v. with LPS. 3 h later, cells recovered from peripheral LNs (pLNs), mesenteric LNs (mLNs) and the spleen (Spl.) were incubated during 2 h with BrefeldinA for determination of intracellular cytokine production. (A) Diagram illustrating the experimental model. (B) Representative IL‐17 dot‐plots for gated Ly‐6C^−^ CD44^hi^ γ/δ T cells recovered from pLNs of LPS‐treated or ‐untreated old WT and old Ifnar^KO^ C57BL/6 mice. (C) Proportions of IL‐17‐producing cells among Ly‐6C^−^ CD44^hi^ γ/δ T cells recovered from pLNs, mLNs and spleen of LPS‐treated or ‐untreated old WT and old Ifnar^KO^ C57BL/6 mice. Data are expressed as mean ± SEM. Results, from at least three independent experiments, were assessed using a two‐tailed unpaired Student's *t*‐test (*p < 0.05; **p < 0.01; ***p < 0.001; ****p < 0,0001, ns, not significant). Each dot on figure panels represents individual mice.

Considering the previous findings, we next examined whether type I IFNs could impact the readiness of innate‐like γ/δ T‐cell subset to respond to LPS challenge with age. To address this issue, 18‐month‐old WT and Ifnar^KO^ mice were intravenously injected with LPS. 3 h later, IL‐17 production by Ly‐6C^−^ CD44^hi^ γ/δ T cells recovered from pLNs, mLNs, and the spleen was analyzed (Figure 6A). As shown in Figure 6B,C, we observed that in all studied SLOs, the proportion of IL‐17‐producing innate‐like γ/δ T cells in response to LPS was significantly lower in aged Ifnar^KO^ mice than in old WT mice. These results confirm that type I IFNs may amplify the IL‐17 production capacity of peripheral γ/δ T cells with age.

## 4 Discussion

Aging is a complex process that impacts multiple physiological functions, including immunity. In this study, we show that the peripheral γ/δ T‐cell compartment undergoes profound age‐associated changes. In particular, aging is characterized by a reshaping of this compartment, most notably through the expansion of IL‐17‐producing innate‐like γ/δ T cells within secondary lymphoid organs (Figure 1). This finding extends previous work by Chen et al. (2019), which focused on peripheral lymph nodes and reported an age‐related increase in the proportion of IL‐17‐producing Vγ6^+^ T cells. Importantly, our study extends these findings by showing that aging not only promotes the expansion of IL‐17‐producing γ/δ T cells but also enhances their functional capacity to secrete IL‐17 (Figures 2 and 3). Mechanistically, we demonstrate that this age‐associated functional enhancement arises from type I IFNs‐mediated downregulation of Foxo1 expression in γ/δ T cells.

One possible hypothesis is that aging might modulate γ/δ T‐cell homeostasis by altering the local availability of certain cytokines, notably IL‐7. There is currently no direct evidence that IL‐7 production is modulated with age (Fry and Mackall 2005; Becklund et al. 2016). However, age‐related declines of IL‐7–consuming lymphocyte populations, particularly the marked reduction in naïve α/β T‐cell numbers within SLOs (Goronzy and Weyand 2017; Durand et al. 2024), may lead to a decreased pool of IL‐7Rα‐expressing T cells (Kim et al. 2006). This reduction could, in turn, lower overall IL‐7 consumption and thereby could increase its availability within SLOs. Because IL‐7 has been shown to preferentially promote the expansion of IL‐17‐producing γ/δ T cells in pLNs (Michel et al. 2012), and that in vivo IL‐7 neutralization strongly impacts the proliferation of this γ/δ T‐cell subset (Chen et al. 2019), the age‐related expansion of IL‐17‐producing γ/δ T cells might partly reflect altered IL‐7 dynamics in aged lymphoid tissues. Interestingly, in the intestine, we also observed an age‐associated increase in the proportion of IL‐7R‐expressing innate γ/δ T cells (Figure S1). Further investigations will be required to fully address this hypothesis.

A decline in Foxo1 expression with age has been previously reported in human CD8 α/β T cells (Delpoux et al. 2021). More recently, we observed that Foxo1 expression is down‐regulated in both CD4 and CD8 α/β T cells from aged mice compared with young controls (Durand et al. 2024). Here, we extend these findings by showing that Foxo1 expression also declines with age in peripheral γ/δ T‐cell subsets, particularly in Ly‐6C^−^ CD44^hi^ γ/δ T lymphocytes, a population exhibiting innate‐like properties (Figure 4). Notably, this reduction in Foxo1 expression correlates with a marked expansion of this subset among γ/δ T cells across all examined secondary lymphoid organs and, strikingly, with their increased capacity to produce IL‐17. Foxo1 has been described as a key regulator of NKT‐cell effector lineage commitment, with Foxo1 deficiency biasing differentiation toward IL‐17–producing NKT cells at the expense of IFN‐**γ**‐producing subsets (Tao et al. 2019). Similarly, we previously reported that Foxo1 acts as a direct negative regulator of the RORγt‐Th17 differentiation program in CD4 α/β T cells (Lainé et al. 2015). Consistent with these findings, age‐associated Foxo1 downregulation is linked to an increased proportion of Th17 CD4 T cells (Ouyang et al. 2011; Lim et al. 2014), as well as enhanced IL‐17 secretion by liver‐resident NKT cells (Stout‐Delgado et al. 2009). Collectively, these observations support the notion that Foxo1 expression may also regulate peripheral γ/δ T‐cell effector functions, and that its age‐associated downregulation in Ly‐6C^−^ CD44^hi^ γ/δ T lymphocytes may contribute to their enhanced capacity to produce IL‐17.

Aging is associated with a state of low‐grade chronic inflammation, characterized by a systemic elevation of pro‐inflammatory cytokines in the absence of overt infection (Fulop et al. 2023). This phenomenon, commonly referred to as “inflammaging,” is thought to contribute to the pathogenesis of numerous age‐related diseases, including cardiovascular disease, neurodegeneration (Yousefzadeh et al. 2021; Carrasco et al. 2022), impaired immune responses to infections (Stout‐Delgado et al. 2009), and reduced efficacy of cancer immunotherapies (Bouchlaka et al. 2013). IL‐6, TNF‐α, and IL‐1 are classical pro‐inflammatory cytokines implicated in the process of inflammaging (Baechle et al. 2023). In addition, type I interferons, particularly IFN‐α, are increasingly recognized as important contributors to this age‐associated inflammatory state (Rasa et al. 2022). Although IFN‐α is classically recognized for its antiviral and immunostimulatory roles (Le Bon et al. 2003; Le Bon et al. 2001), persistent low‐level type I IFN signaling with age has been linked to T cell senescence (Abbas and Akbar 2021). Moreover, chronic IFN‐α exposure has been shown to inhibit telomerase activity and promote telomere erosion in both stimulated CD4 T cells (Reed et al. 2004) and CD8 T cells (Lanna et al. 2013). In aged mice, aberrant activation of the cGAS–STING signaling pathway has been shown to drive age‐associated type I IFN responses, thereby contributing to a pro‐inflammatory milieu and sustaining the low‐grade systemic inflammation characteristic of “inflammaging” (Gulen et al. 2023). Interestingly, we recently demonstrated that sustained IFN‐α exposure during aging promotes Foxo1 downregulation in α/β T cells both in vitro and in vivo, contributing to their exhaustion and to the functional aging of the α/β T‐cell compartment (Durand et al. 2024). Consistent with these findings, we now show that type I IFNs are also implicated in the age‐related downregulation of Foxo1 in peripheral γ/δ T cells, enhancing the IL‐17–producing capacity of Ly‐6C^−^ CD44^hi^ γ/δ T cells recovered from pLNs, mLNs and the spleen (Figure 5). Collectively, these results suggest that this mechanism may help sustain age‐associated low‐grade inflammation, thereby reinforcing the “inflammaging” vicious cycle.

In previous studies, we and others have shown that Ly‐6C^−^ CD44^hi^ γ/δ T cells reside within SLOs, where their long‐term retention is primarily mediated by macrophage subsets (Audemard‐Verger et al. 2017; Zhang et al. 2016). Furthermore, we demonstrated that these innate‐like γ/δ T cells can rapidly produce IL‐17 in vivo in response to LPS challenge (Audemard‐Verger et al. 2017). In this context, LPS may activate them either directly or indirectly via induction of IL‐23 and IL‐1β production by neighboring macrophages, consistent with the observation that RORγt^+^ γ/δ T cells express IL‐23R and produce IL‐17 in response to IL‐1β and IL‐23 independently of TCR engagement (Sutton et al. 2009). Our present data clearly demonstrate that the capacity of Ly‐6C^−^ CD44^hi^ γ/δ T lymphocytes from SLOs to mount a rapid in vivo response upon LPS challenge increases with age (Figure 3), and that this results from type I IFNs‐induced Foxo1 down‐regulation (Figure 6). These findings are particularly noteworthy given that type I interferon‐induced downregulation of Foxo1 contributes substantially to α/β T‐cell exhaustion and dysfunction in aged mice (Durand et al. 2024). Despite this, resident γ/δ T cells and macrophages may still provide a critical barrier against systemic pathogen spread within SLOs through rapid innate responses. Conversely, the age‐related expansion of IL‐17‐producing γ/δ T cells might influence the balance toward a γ/δ17 lineage, which has been associated with pro‐tumorigenic activities in several mouse models. IL‐17–producing γ/δ T cells have indeed been implicated in tumor growth and metastasis in spontaneous models of breast cancer and pancreatic intraepithelial neoplasia (Coffelt et al. 2015; McAllister et al. 2014), as well as in multiple transplantable tumor models (Ma et al. 2014; Rei et al. 2014; Carmi et al. 2011). More recently, the age‐dependent accumulation of IL‐17‐producing γ/δ T cells has been associated with the development of lung melanoma metastases in mice (Cheng et al. 2020; Duan et al. 2024). However, these associations should be interpreted with caution, as they do not establish a direct causal link between age‐related γ/δ17 expansion and tumor susceptibility. Alternatively, the enrichment of IL‐17‐producing γ/δ T cells in aged tissues could also reflect a compensatory or adaptive response aimed at maintaining barrier integrity or enhancing local immune surveillance during aging.

Collectively, these findings suggest that γ/δ T‐cell remodeling with age may have context‐dependent effects, potentially supporting antimicrobial defense while modifying the tissue environment in ways that could influence tumor dynamics. In this context, the age‐related decline in Foxo1 may represent one molecular pathway through which type I IFNs and other extrinsic cues shape γ/δ T‐cell function during aging. Future studies will be required to clarify the functional consequences of these changes and to determine whether targeting the type I IFNs–Foxo1 axis might preserve beneficial γ/δ T‐cell responses while minimizing potential detrimental effects.

## Author Contributions

A.D., S.P., and C.W. performed data acquisition and analysis. E.X. performed bioinformatic analyses and data interpretation. A.L.B. provided resources and contributed to revising the manuscript for important intellectual content. C.A. performed bioinformatic analyses and data interpretation and contributed to drafting the manuscript. B.L. contributed to the conception and design of the study and to writing the manuscript. B.M. conceived and designed the study, performed data acquisition, analysis, and interpretation, and wrote the manuscript.

## Funding

This work was supported by a grant from the “Fondation pour la Recherche Médicale” (FRM team number EQU202103012662).

## Conflicts of Interest

The authors declare no conflicts of interest.

## Supporting information


**Figure S1:** Age‐associated single‐cell characterization of γ/δ T‐cell subsets in lung, colon and ileum of C57BL/6 mice.
(A) UMAP projection showing the single‐cell distribution of lung, colon and ileum γ/δ T cells from C57BL/6 mice, based on bioinformatic analyses used to define cell clusters (Zhang et al.). (B) Dot plot illustrating marker gene expression across γ/δ T‐cell subsets. The color denotes average expression levels, and dot size indicates the percentage of cells expressing each marker. (C) Proportions of defined γ/δ T‐cell clusters among total γ/δ T cells in the lung, colon and ileum as a function of age in C57BL/6 mice.
**Figure S2:** Comparison of the peripheral γ/δ T‐cell compartment in aged WT and IfnarKO mice.
Absolute numbers of γ/δ T‐cell subsets, categorized by their Ly‐6C and CD44 expression, recovered from pLNs, mLNs and the spleen of old (18‐month‐old) WT and Ifnar^KO^ C57BL/6 mice. Data are expressed as mean ± SEM. Results, from at least three independent experiments, were assessed using a two‐tailed unpaired Student's *t*‐test (*p < 0.05; **p < 0.01; ***p < 0.001; ****p < 0,0001, ns, not significant). Each dot on figure panels represents individual mice.

## Data Availability

Bioinformatic data that support the findings of this study are openly available in the following study (Zhang et al. 2025) at DOI: 10.1126/science.adn3949. Raw FASTQ files, processed count matrices, cell metadata, and gene metadata can be downloaded from NCBI Gene Expression Omnibus under accession number GSE247719. All other data generated in this study are available within the article and its Supporting Information files or from the corresponding author upon reasonable request.
