# Supplementary material for: Aging Reshapes γ/δ T‐Cell Immunity Through a Type I Interferon–Foxo1 Axis
Source: Aging Cell. 2026 Jan 20;25(2):e70389. doi: 10.1111/acel.70389 (PMC12820350; doi:10.1111/acel.70389)
Supplement: Supplementary file 1 — Figure S1: Age‐associated single‐cell characterization of γ/δ T‐cell subsets in lung, colon and ileum of C57BL/6 mice. (A) UMAP projection showing the single‐cell distribution of lung, colon and ileum γ/δ T cells from C57BL/6 mice, based on bioinformatic analyses used to define cell clusters (Zhang et al.). (B) Dot plot illustrating marker gene expression across γ/δ T‐cell subsets. The color denotes average expression levels, and dot size indicates the percentage of cells expressing each marker. (C) Proportions of defined γ/δ T‐cell clusters among total γ/δ T cells in the lung, colon and ileum as a function of age in C57BL/6 mice. Figure S2: Comparison of the peripheral γ/δ T‐cell compartment in aged WT and IfnarKO mice. Absolute numbers of γ/δ T‐cell subsets, categorized by their Ly‐6C and CD44 expression, recovered from pLNs, mLNs and the spleen of old (18‐month‐old) WT and IfnarKO C57BL/6 mice. Data are expressed as mean ± SEM. Results, from at least three independent experiments, were assessed using a two‐tailed unpaired Student's t‐test (*p < 0.05; **p < 0.01; ***p < 0.001; ****p < 0,0001, ns, not significant). Each dot on figure panels represents individual mice. [file ACEL-25-e70389-s001.docx]

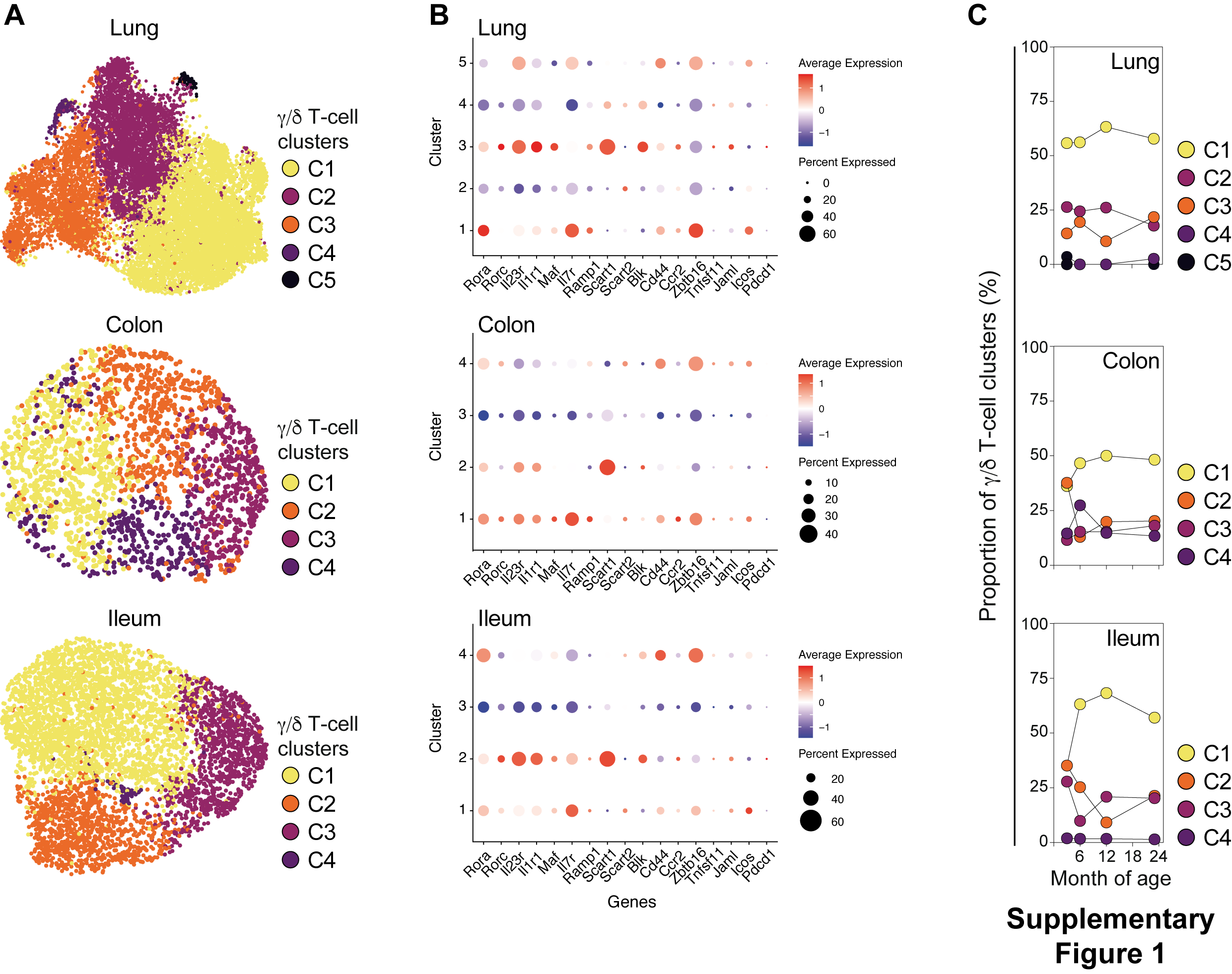


**Supplementary Figure 1: Age-associated single-cell characterization of γ/δ T-cell subsets in lung, colon and ileum of C57BL/6 mice.**

(A) UMAP projection showing the single-cell distribution of lung, colon and ileum γ/δ T cells from C57BL/6 mice, based on bioinformatic analyses used to define cell clusters (Zhang et al.). (B) Dot plot illustrating marker gene expression across γ/δ T-cell subsets. The color denotes average expression levels, and dot size indicates the percentage of cells expressing each marker. (C) Proportions of defined γ/δ T-cell clusters among total γ/δ T cells in the lung, colon and ileum as a function of age in C57BL/6 mice.

**
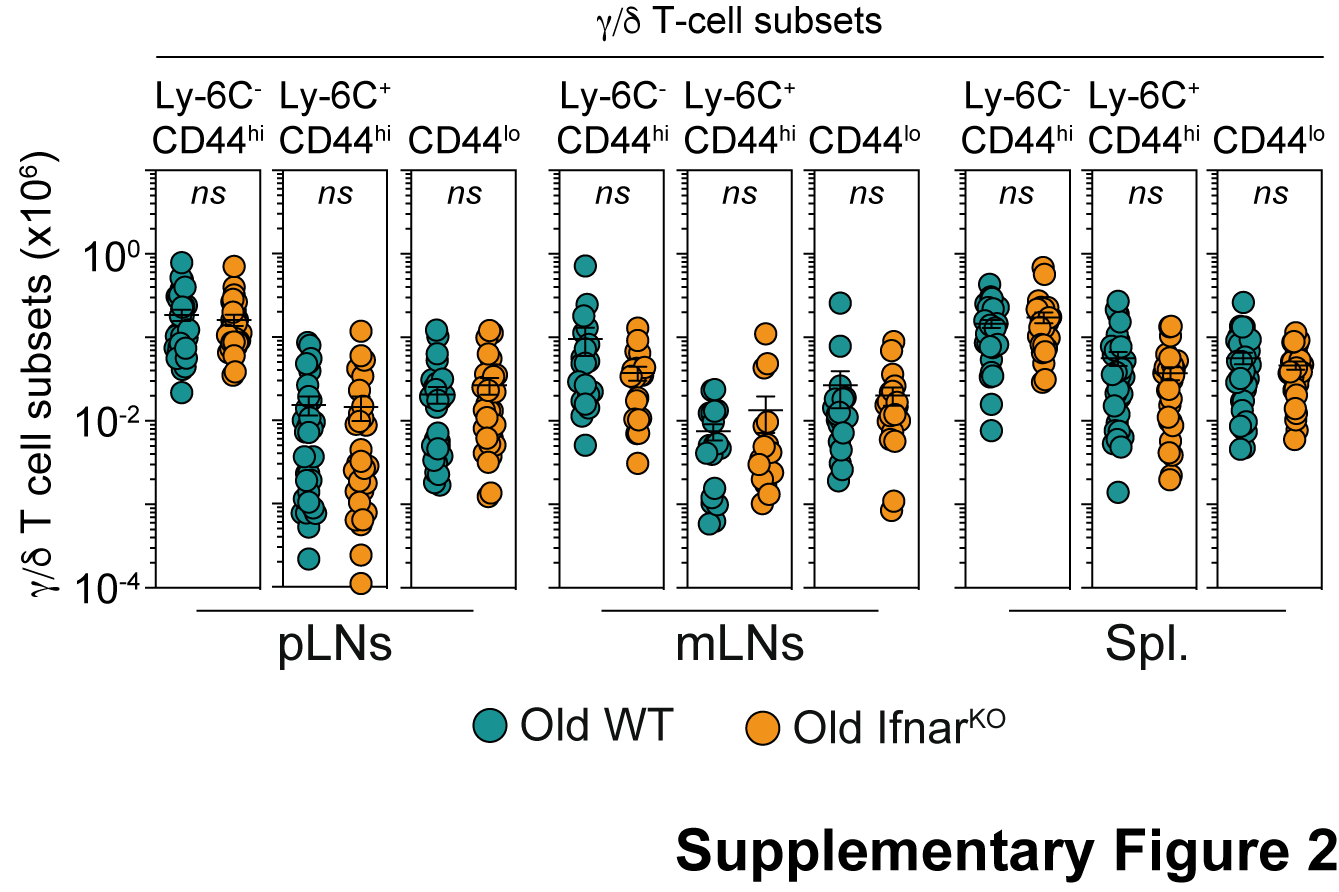
**

**Supplementary Figure 2: Comparison of the peripheral γ/δ T-cell compartment in aged WT and Ifnar^KO^ mice**

Absolute numbers of γ/δ T-cell subsets, categorized by their Ly-6C and CD44 expression, recovered from pLNs, mLNs and the spleen of old (18-month-old) WT and Ifnar^KO^ C57BL/6 mice. Data are expressed as mean ± SEM. Results, from at least three independent experiments, were assessed using a two-tailed unpaired Student’s *t*-test (**p* < 0.05; ***p* < 0.01; ****p* < 0.001; *****p* < 0,0001, ns, not significant.). Each dot on figure panels represents individual mice.
